# Energy and Nutrient Issues in Athletes with Spinal Cord Injury: Are They at Risk for Low Energy Availability?

**DOI:** 10.3390/nu10081078

**Published:** 2018-08-13

**Authors:** Katherine Figel, Kelly Pritchett, Robert Pritchett, Elizabeth Broad

**Affiliations:** 1 Central Washington University, Ellensburg, WA 98926, USA; Kelly.pritchett@cwu.edu (K.P.); Robert.pritchett@cwu.edu (R.P.); 2 United States Olympic Committee, Chula Vista, CA 91915, USA; Elizabeth.broad@usoc.org

**Keywords:** spinal cord injury, athlete, energy availability, nutrient deficiency, low energy availability, bone mineral density, para athlete, menstrual dysfunction, Female Athlete Triad, Relative Energy Deficiency in Sports (RED-S)

## Abstract

Low energy availability (LEA) and nutrient intake have been well studied in able-bodied athletes, but there is a lack of research examining these issues amongst athletes with spinal cord injury (SCI). To date, there have been no studies that have examined energy availability (EA) amongst this population. Furthermore, athletes with SCI may experience unique challenges around nutrition that may increase their risk of LEA. This review will evaluate the literature and assess whether this population is at risk for LEA. Due to the limited research on this topic, sedentary individuals with SCI and para athletes were also included in this review. Review of the current literature suggests that athletes with SCI may be at an increased risk for LEA. While research examining EA and risk of LEA in athletes with SCI is lacking, the number of athletes with SCI continues to increase; therefore, further research is warranted to assess nutrient and energy needs and their risk to this population.

## 1. Introduction

Energy and nutrient availability have been widely studied in able-bodied athletes; however, there is a lack of research examining energy availability (EA) and nutrient intake in para athletes, including athletes with a spinal cord injury (SCI). Para athletes are defined as athletes with physical disabilities [1,2,3]. Athletes who experience an SCI are a specific group within the para athlete group. While there is no research specially looking at EA amongst athletes with SCI, some studies assessing energy intake and expenditure have suggested that this population may be at increased risk for LEA. 

Low energy availability (LEA) can lead to menstrual dysfunction and low bone mineral density (BMD) in able-bodied athletes. These conditions are included in the Female Athlete Triad (Triad) and are characterized on a spectrum ranging from optimal health to a disease state. The Triad fits within the more recent, broader, and more comprehensive term for the condition called Relative Energy Deficiency in Sport (RED-S) [4,5]. LEA and components of the Triad and RED-S can lead to decreased athletic performance and serious short and long-term health consequences. This highlights the need for early detection, diagnosis, and treatment of these medical conditions amongst male and female athletes [4,5].

Athletes with SCI have differing energy requirements and bone densities compared to the able-bodied population. This makes it more challenging to identify whether this group is at risk for LEA, and yet this research is even more vital given this lack of knowledge and growing number of athletes with SCI [1]. While menstrual dysfunction and low BMD may be caused by LEA in the able-bodied population, these conditions may be related to the athlete’s disability rather than LEA in the para athlete population, including athletes with SCI [1,2].

Health issues associated with SCI may also put these athletes at a greater risk for LEA. Pain associated with the injury may decrease appetite, while medications used to manage pain may cause constipation or nausea. Kidney and bladder infections are more common amongst athletes with SCI compared to able-bodied athletes. The use of antibiotics to treat or prevent these infections may cause diarrhea or a decrease in gut bacteria, which could result in compromised gut health and nutrient absorption. Challenges around grocery shopping and preparing food due to physical limitations may also influence energy intake. In addition, athletes with SCI may experience difficulties swallowing, leading to decreased food intake. Finally, individuals with SCI may increase fiber intake to regulate bowels, although excessive fiber intake may decrease overall energy intake, since it has a slower gastric emptying time.

Oftentimes, when an individual experiences an SCI, they receive nutrition counseling focusing on weight management, since their energy needs are decreased compared to those of an able-bodied individual. When they become an athlete however, their energy needs increase compared to sedentary individuals with SCI. Given that there is a paucity of research around the nutritional needs of athletes with SCI, these athletes may not receive proper education or guidance around their adjusted calorie needs and thus may be at increased risk for LEA.

The number of athletes participating in para sport is increasing with a record number of athletes participating in the 2018 Winter Paralympics in PyeongChang [6]. In addition, the number of athletes participating in the 2016 Summer Paralympics in Rio was over 4300 [7]. Para athletes, including athletes with SCI, may be at risk for inadequate dietary intakes, and thus more research is needed amongst this population [1,2,3,8,9,10,11]. This review evaluates the literature examining EA, LEA, and related conditions among athletes with SCI.

## 2. Materials and Methods 

A review of literature was conducted between January and May of 2018 using PubMed. Search terms included BMD, EA, nutrient intake, SCI, para athlete, total energy expenditure (TEE), energy intake (EI), exercise energy expenditure (EEE), LEA, and menstrual function in reference to athletes with SCI. Persons with an SCI include people with full or partial paralysis to parts of their body due to an injury to the spinal cord. Paraplegia and tetraplegia are the result of a complete or incomplete injury to the spine. Original research, reviews, and relevant books published in 1985 or later were included in the search. To date, we have been unable to find any research studies examining EA in athletes with SCI. In addition, there was limited data specifically related to athletes with SCI (who are defined as someone who has experienced an injury to their spinal cord and now participates in sports). Therefore, research using sedentary individuals with SCI and para athletes (who are defined as athletes with a disability that may be SCI but could also include amputation, spina bifida, visual impairment, cerebral palsy, or acquired brain injury) was also included.

## 3. Results

### 3.1. Energy

#### 3.1.1. Energy Intake

Collection tools such as food journals and food frequency questionnaires have limitations and great difficulties in accurately capturing energy intake and thus create challenges around accurate assessment and data collection [12]. Participant-recorded food journals may reflect underreporting of energy and nutrient intake, as subjects tend to underestimate portion sizes or fail to include all food consumed during the collection timeframe. It is estimated that amongst athletes, underreporting accounts for 10–45% of total energy expenditure [13]. Most studies included in this review paper used a 24 h or 3-day food record as their collection method and did not correct for underreporting amongst subjects. Disadvantages to this method include high subject burden and cooperation, and may cause altered diet behaviors due to act of recording food intake [14]. While short term food journals are primarily used, it is estimated that a diet record may need to be kept for 27–35 days in order to accurately assess energy intake in male and female subjects [14]. In addition, many studies assessing energy intake amongst para athletes, including athletes with SCI, use food journals while athletes are at training camp facilities where meals are provided. While this may enable more accurate estimation of nutrient intake, it may provide an inaccurate picture of what the athlete is consuming at home when food choices may be different, although Krempien et al. [9] found no difference in overall energy intake when athletes kept a 3-day food journal at home versus at training camp. Obstacles associated with grocery shopping, food preparation, and cooking may provide additional challenges amongst athletes with SCI who have limited accessibility. These obstacles may greatly impact athletes’ food choices and may put them at greater risk of experiencing inadequate food and nutrient intake and thus LEA. Dysphagia (difficulty swallowing) and slower gastric emptying are conditions some athletes with SCI experience; they can also increase the risk of inadequate energy and fluid intake [3]. Medications commonly used by individuals with SCI may cause unwanted side effects such as nausea, gastrointestinal upset, poor sleep patterns, and changes in gut bacteria and appetite. These side effects may influence diet, energy intake, and nutrient absorption of athletes with SCI [3].

Studies have found that energy intakes amongst male para athletes vary greatly, ranging from 23–64 kcal/kg/day [11]. Despite lower energy needs, athletes with SCI may still be consuming too few calories [15,16]. Madden et al. [16] found that elite male and female wheelchair athletes consumed on average 2092 kcal/day and 1602 kcal/day, respectively. Similar trends were observed in male and female athletes with SCI with average energy intake found to be comparable to or lower than the energy intake recommendations of sedentary, able-bodied individuals [8,9,10].

While Grams et al. [11] found a higher average energy intake amongst male wheelchair basketball players (2673 kcal/day) compared with other studies, energy intakes ranged considerably between individuals from 1597 to 3651 kcal/day. This suggests that there is a wide variation in energy intake amongst this population even within the same sport [11]. This variation is likely due to differences in body weight, disability, and injury level. While more research needs to be done, actual energy intake amongst athletes with SCI is considerably lower compared to recommendations for able-bodied athletes. Given that male and female athletes with SCI have increased physical activity levels, their energy needs are likely greater than able-bodied sedentary individuals and thus their intake may be too low [11,17].

#### 3.1.2. Total Energy Expenditure (TEE)

Total energy includes resting metabolic rate (RMR), thermic effect of feeding (TEF), and physical activity. RMR measures the energy that your body burns while at rest. Male and female athletes with SCI have lower energy requirements compared to able bodied athletes [3,17,18,19]. In addition to the amount of physical activity, level and severity of spinal cord lesion also play a role in determining overall energy expenditure of an individual. TEE studies have primarily been conducted in able-bodied persons and athletes, while limited studies have assessed energy expenditure in the SCI population. Furthermore, most work has been done in clinical settings on sedentary individuals with SCI, therefore little is known regarding the energy cost of wheelchair-based activities. Table 1 shows studies that have examined energy expenditure in para athletes including athletes with SCI. It should be noted that in order to measure TEE, fat free mass (FFM) needs to be assessed. Most studies used dual-energy x-ray absorptiometry to assess body composition.

#### 3.1.3. Resting Metabolic Rate (RMR)/Basal Metabolic Rate (BMR)

It has been suggested by Mollinger et al. [21] that individuals with SCI may have a 12–27% lower basal metabolic rate (BMR) when compared to able-bodied individuals [21]. In Mollinger’s study, BMR was measured on three consecutive days using 10-min samples of expired air shortly after the subject was awakened in the morning. The variance in BMR in individuals with SCI is correlated with the level of lesion and whether the injury is a complete or incomplete lesion [21]. A lower BMR is associated with a higher level of lesion. A complete injury is also associated with a lower BMR compared to an incomplete SCI, which allows for limited nerve signaling from the spinal cord to brain and possible limited movement and sensation below the injury site. The greatest difference in BMR compared to the able-bodied population is believed to be due to decreased muscle mass and increased adipose tissue in individuals with SCI. Furthermore, studies have suggested that when adjusted for lean body tissue, there is no difference or possibly even a greater RMR in individuals with SCI compared with a control group of able-bodied individuals [17,18,20]. Interestingly, Pelly et al. [20] found that when adjusted for lean tissue mass (LTM), RMR was actually higher in athletes with SCI compared to controls. This suggests that athletes with SCI were using more calories per kg of LTM. These results may be due to the greater metabolic activity of the viscera compared to skeletal muscles at rest [20]. Both Pelly et al. [20] and Buchholz et al. [18] measured RMR with subjects in a reclined position, refraining from caffeine and exercise for 24 h prior to testing and following a 12 h fast. Females were self-reported to be in the follicular phase of their menstrual cycle. Buchholz et al. [18] used male and female wheelchair athletes, while Pelly et al. [20] used male athletes with SCI as subjects.

Accurately measuring TEF is very challenging. This review looked at two studies examining differences in TEF in the SCI population compared to the able-bodied population. Both Aksnes et al. [22] and Buchholz et al. [18] found no significant difference in TEF between individuals with SCI and able-bodied controls. Aksnes et al. [22] measured TEF in the morning after subjects had gone through an overnight fast of 12–14 h. Expired gas was collected from subjects using a mouth piece for measurements of ventilation. Respiratory gas exchanges were measured using a pneumotachograph and paramagnetic and infrared analyzers for oxygen and carbon dioxide, respectively. Expired gas was collected for 30 min in the resting state and for 7 min at the end of each 15-min time segment for 2 h post prandial. Venous blood samples were taken before and every 15 min post prandial to determine plasma concentrations of glucose and insulin. The meal was in a liquid form and was comprised of 52% carbohydrate, 37% fat, and 11% protein. The control group received only water in the same corresponding volume of the liquid meal provided to the other group.

Buchholz et al. [18] also measured TEF using indirect calorimetry for 2 h post prandial. Subjects reported for the study following a 12 h fast. Expired air was measured using a ventilated canopy for 60 min with the last 40 min of the data used. TEF was then measured with indirect calorimetry for 120 min after the plateau blood sample and following consumption of a mixed liquid meal consisting of 55% carbohydrate, 30% fat, and 15% protein. The dose of the meal was calculated to be 30% of RMR for each subject.

#### 3.1.4. Exercise Energy Expenditure (EEE)

It is estimated that male and female athletes with SCI have 25–75% lower energy needs compared to able-bodied athletes [17]. Expended energy depends on the sport and level of injury. For example, male wheelchair basketball players are estimated to expend 75% of the calories expended by able bodied basketball players. An even greater energy discrepancy is found in men’s rugby, where it’s estimated that wheelchair rugby players expend only 26% of the calories expended by able bodied male rugby players [17].

Numerous tools have been used to assess energy expenditure amongst individuals with SCI, including indirect calorimetry, heart rate monitoring, accelerometers, and energy expenditure questionnaires including the Physical Activity Recall Assessment for People with Spinal Cord Injury (PARA-SCI) and the Leisure Time Physical Activity Questionnaire for People with Spinal Cord Injury LTPAQ-SCI [23,24]. However, there are limitations surrounding these methods when applying them to athletes with SCI [2]. As a result, there is insufficient research to determine a validated gold standard for assessing energy expenditure in athletes with SCI [20,23].

In addition, individuals with SCI, particularly those with a high level spinal lesion, may have reduced sympathetic nervous system (SNS) availability compared to able-bodied individuals. The SNS is involved with hepatic glycogenolysis and gluconeogenesis, and thus may impact the metabolism of athletes with SCI [17]. Individuals with SCI also have reduced muscle mass available for exercise capacity [17,19]. This, combined with a decrease in SNS activity and lower VO_2peak_ and peak power output, can result in reduced exercise capacity compared with able-bodied controls, suggesting that these athletes may expend less energy compared to able-bodied athletes doing the same activity [19].

Some studies have measured energy expenditure while athletes are living at training camps. Distances between living and training quarters, as well as terrain, can all significantly impact the energy expenditures of these athletes, since the calories expended getting to these locations may be greater compared to that of able-bodied individuals. Thus, energy expenditure may differ drastically depending on the athlete’s setting. Mode of ambulation and completeness of injury may also differ, which influences energy expenditure and makes it harder to assess energy expenditure in athletes with SCI. Although further research is warranted, non-exercise activity thermogenesis (NEAT) may be greater in people with SCI given that it may be more challenging to accomplish daily activities, while energy cost during structured exercise may be lower than what is observed in able bodied athletes.

#### 3.1.5. Energy Availability

Research has yet to examine EA in athletes with SCI. However, some literature looking at energy intake or energy expenditure suggests that both male and female athletes with SCI may demonstrate risk factors for LEA [1,3,25]. EA is calculated as total energy intake (kcals) minus exercise energy expenditure (kcals) divided by kilograms (kg) of fat free mass (FFM). Optimal energy availability in female athletes is believed to be >45 calories per kilogram of FFM. Reduced energy availability in this population is defined in the literature as <45 kcals/kg/FFM, and LEA is defined as less than 30 calories per kilogram of FFM per day, although may be lower amongst males [4,5,26,27,28]. These cutoffs may not predict associated consequences amongst all athletes, including males, and have yet to be validated in athletes with SCI [5]. Thus, this equation and reference ranges may be inappropriately applied to athletes with SCI due to their lower active muscle mass, decreased mobility, and reductions in the sympathetic nervous system due to paralysis [17,18].

When an athlete experiences reduced EA, subclinical menstrual disorders and low BMD may occur [29]. Identifying whether an athlete with SCI has LEA is challenging, since these conditions may be present regardless of EA. Melin et al. [26] estimated that 63% of elite, able-bodied female endurance athletes were found to have either low (<30 kcal/kg FFM/day) or reduced EA (<45 kcal/kg) over a 7-day study period [26]. However, the incidence of LEA amongst male and female athletes with SCI has yet to be determined.

LEA can be acute, chronic, or intermittent. There is currently no established guideline pertaining to the length of time an athlete must be experiencing LEA in order to be considered at “chronic” LEA. For example, many research studies use short term/acute tools such as 24 h or 7-day food journals to collect data, which may over or under estimate energy intake and result in inaccurate conclusions around long term energy availability amongst athletes. However, even a short timeframe of LEA is believed to be disruptive to metabolic substrate and hormone function. Loucks and Thuma [30] found that after only 5 days of LEA (<30 kcal/kg FFM/day), adult females experienced reductions in blood glucose levels and hypothalamic pituitary-axis hormones [30].

Gorgey et al. [15], using indirect calorimetry and four 5-day participant recorded food diaries, suggested that calorie intake amongst sedentary males with SCI using either a manual or power wheelchair was significantly lower than estimated total energy expenditure (TEE).

#### 3.1.6. Nutrition Knowledge and Behaviors

Poor nutrition knowledge has been suggested to be a risk factor for inadequate energy intake amongst able-bodied athletes [31,32]. Zawila et al. [31] found that 83.3% of abled bodied female cross-country runners responded yes to the statement “Does your knowledge of nutrition affect how you eat?”. While it is unknown what effect this has on their nutrition, 91.7% of subjects in the same study strongly agreed or agreed with the statement “Learning facts about nutrition is the best way to achieve favorable changes in food habits”. Nutrition knowledge amongst athletes with SCI may be lacking. Research suggests that para athletes may be lacking in nutrition knowledge [25,33]. Eskici and Ersoy [25], using a 76-question nutrition knowledge questionnaire, found that the majority of female wheelchair basketball players in their study had inadequate nutrition knowledge, particularly pertaining to sport nutrition-specific information such as nutrition recommendations to support recovery and hydration of athletes.

Nutrition support provided to para athletes varies between countries and is still relatively new, although it is increasing across the globe. In the USA, para athletes and many able-bodied sports are not supported by the National Collegiate Athletic Association (NCAA), and hence these teams and athletes may not receive the nutritional resources and support that other athletes receive [2]. The lack of knowledge amongst the population highlights the need for access to a registered sports dietitian to improve nutrition and decrease the risk for LEA.

### 3.2. Risk for Disordered Eating

#### 3.2.1. Attitudes about Eating

More research is needed regarding the eating attitudes and behaviors of athletes with disabilities, as there is no research looking at the prevalence of eating disorders amongst athletes with SCI. However, because athletes with SCI may have lower energy needs, this population may be at risk for disordered eating [1,34]. Krempien et al. [34] found that male athletes with SCI exhibited strong tendencies towards cognitive dietary restraint, while female athletes with SCI exhibited similar scores to able-bodied populations. Both male and female athletes with SCI showed lower scores for disinhibition and hunger compared to able-bodied individuals, suggesting that they may monitor body weight for sport. While the reason for this is not currently understood, it should be noted that this characteristic could put this population at risk for LEA [34].

#### 3.2.2. Leanness in Sports

In the able-bodied population, athletes competing in a sport emphasizing leanness may be at increased risk of LEA compared with athletes competing in other sports. However, this correlation is not a well-established risk factor amongst para athletes. Krempien et al. [34] suggested that elite athletes with SCI display behaviors that may be associated with LEA, such as strong tendencies towards dietary restraint and restriction of energy intake (Table 2). Blauwet et al. [35] found that there was no difference around risk for low energy intake amongst male and female para athletes who competed in sports emphasizing leanness compared to other sports. This suggests that para athletes may be at risk for LEA regardless of sport. In addition, 40% of subjects reported currently trying to lose weight, and 61% reported attempting to change their body composition to improve their performance [35]. While more research is warranted, the responses show that athletes with SCI may be at an increased risk for LEA.

### 3.3. Nutrients

#### 3.3.1. Macronutrient Intake

Based on the recommendation used for able-bodied athletes, athletes should consume between 3.0–12.0 g of carbohydrate (CHO)/kg of body weight (BW) and 1.2–2.0 g of protein/kg BW [36]. These ranges accommodate variances due to sport, intensity, duration, etc. The literature review found limited studies examining the macronutrient intake of para athletes, and most studies used 24-h food journals (Table 3). Madden et al. [16] found that male and female elite wheelchair athletes had a mean intake of 3.5 g CHO/kg BW. Females consumed less protein at 1.4 g protein/kg BW, while males consumed 1.6 g protein/kg BW. Gerrish et al. [8] and Goosey-Tolfrey et al. [10] found similar results, with wheelchair athletes consuming between 3.1–4.3 g CHO/kg BW, females consuming 1.0–1.1 g protein/kg BW, and males consuming 1.4–1.7 g protein/kg BW. While these technically fall within the appropriate range, carbohydrate intakes for both sexes and protein intake for females are at the lower end of recommendations, which may suggest increased risk of low energy intake.

#### 3.3.2. Micronutrient Intake

Inadequate macronutrient intake may cause athletes with SCI to experience low micronutrient intake [3,8,9,10,11,25]. To the best of our knowledge, with the exception of vitamin D, there is no data looking at actual micronutrient status amongst individuals with SCI. It is, however, well documented that athletes with SCI are at increased risk for vitamin D deficiency [37], and they may be lacking in additional nutrients. The prevalence of vitamin D deficiency or insufficiency ranges from 32–93%, which is high compared to the able-bodied population. Some factors influencing deficiency include immobility, lesion level, the presence of pressure ulcers, and whether the person practices their sport indoors or outdoors [37]. Table 4 includes studies found in the literature review that examined micronutrient intake of para athletes. Krempien et al. [9] found that over 25% of male and female athletes with SCI had micronutrient intakes below the Estimated Average Requirement (EAR) for calcium, magnesium, folate, and vitamin D. Vitamin and mineral supplementation increased overall micronutrient intake amongst men, while supplement use amongst women did not significantly change their nutrient intake. Even when factoring in supplement use, athletes still did not consume adequate amounts of these nutrients [9]. Gerrish et al. [8] found that during winter training camp, over 60% of female athletes with SCI failed to meet the EAR of many micronutrients including vitamin D, vitamin B6, vitamin B12, vitamin C, folate, calcium, iron, magnesium, and zinc. Male athletes displayed similar trends around micronutrient insufficiencies [9,11]. Madden et al. [16] suggested that while para athletes met most of their micronutrient RDAs, females fell short for iron and calcium, while males did not meet the RDA for vitamin A and folate. In addition to deficiencies related to diet, impairments and altered GI function associated with SCI may also put an athlete at greater risk for micronutrient deficiencies compared to able-bodied athletes [3]. Lastly, there are challenges around accurately assessing true micronutrient intake. Studies that use food journals as a collection method may have underreporting issues. The studies that we included in our review utilized this collection method, so it should be noted that this is a limitation to the data presented.

These studies suggest that regardless of whether energy and macronutrient intakes are adequate, athletes with SCI must maximize their diet with high nutrient-dense foods, given their lower energy “budget” compared to able-bodied athletes [2]. However, nutrient-dense foods may be perceived as expensive. Athletes with SCI are not supported by the NCAA, and very few are professional athletes; thus, financial restrictions may also influence food choices and increase the intake of low nutrient-dense foods by athletes with SCI. Inadequate consumption of these nutrients may put an athlete at risk for poor bone health, low energy levels, compromised immunity, and other health concerns associated with these nutrient deficiencies.

### 3.4. Menstrual Dysfunction

Menstrual dysfunction refers to various irregularities including oligomenorrhea, which is defined as experiencing nine or fewer menstrual periods over a 12 month timeframe, or secondary amenorrhea, which is defined as cessation of menses for at least three consecutive months [38]. These definitions are once again used for both the female able-bodied population and those with disabilities.

Menstrual dysfunction can occur when an athlete has LEA (<30 kcal/kg FFM). It is well documented that menstrual dysfunction not only has an impact on reproductive health but can also lead to negative health consequences, including increased risk around the number of cardiovascular risk factors and premature osteopenia and osteoporosis [26,39]. BMD may decrease as the frequency of missed menstrual cycles increases. Menstrual dysfunction was identified as an independent predictor of bone stress injuries (BSI) when using the 2014 Female Athlete Triad Cumulative Risk Assessment [40]. Furthermore, prevalence of stress fractures is 2–4 times greater in amenorrheic athletes than eumenorrheic athletes [40].

The majority of research suggests that while changes in menstruation may occur after the acute phase post SCI, no chronic disruptions in menstrual function have been noted amongst females with SCI [1,38,41,42]. Stress experienced from the trauma of the injury and dysfunction of the hypothalamic-pituitary axis as a result of the injury may occur. In turn, acute low levels of sex hormones can temporarily stop menses [1]. Females with SCI typically resume menstruation 5 or 6 months following their injury (on average), while many athletes experience no disruptions in menstruation whatsoever [1,38,42]. Adolescent females who sustained a SCI prior to menarche experience the onset of menses at an age similar to that of their mothers and to individuals with SCI who were injured after menarche [38]. In addition, the level of lesion amongst females with SCI does not seem to influence length or duration of menses, and fertility is believed to be unimpaired in individuals with SCI [38,42].

There is currently limited research examining low energy availability and menstrual health amongst athletes with SCI. However, Blauwet et al. [35] found that 32% of pre-menopausal female para athletes (including athletes with SCI and other conditions) reported oligomenorrhea. While more research is warranted, this suggests that female para athletes, including athletes with SCI, may have a high prevalence of menstrual dysfunction. Whether this is due to LEA or other reasons is yet to be determined.

Recent data suggests that 27.6% of US and 30.0% of UK women of reproductive age use hormonal contraceptives [43,44]. Contraceptive use to manipulate or delay menstruation may be a tool implemented by female athletes, since menstruation and menstrual symptoms may be perceived as barriers to physical activity [45]. In a recent study, 50% of elite female athletes believed that their menstrual cycle affected training and performance [46]. Martin et al. [47] found that 50% of elite able-bodied athletes were currently using hormonal contraceptives. The use of hormonal contraceptives may be higher amongst elite athletes compared to that of the general population [48]. The use of contraceptives amongst athletes with SCI is unknown.

Martin et al. [47] found that of the elite athletes using hormonal contraceptives, many reported benefits around the ability to predict and/or manipulate their menses [47]. Schaumberg [45] found that 73% of competitive female athletes reported manipulating their menstrual cycle at least once over the past year. Of that group, 54% reported doing so for sports-related training or competition [45]. While there is no research pertaining to contraceptive use or manipulation of menses amongst athletes with SCI, this rate may be equal or even greater given that menstruation management may be perceived as more challenging or difficult amongst this population. Practitioners should be aware that contraceptive use may mask menstrual dysfunction and make it harder to assess risk of LEA and the Triad amongst athletes with SCI.

### 3.5. Bone Mineral Density

Low BMD places an athlete at increased risk for osteoporosis, in which bones become fragile and brittle [49]. While osteoporosis cannot be diagnosed by *z*-score alone, a BMD *z*-score ≤ −2.0 is defined as “below the expected range for age” for males and females [29,50], while a *z*-score < −1.0 defines low BMD in female athletes in weight-bearing sports [29,39]. However, it is unclear whether these are appropriate diagnostic criteria for people with SCI [1]. In addition, low BMD can increase the risk of bone-related injuries such as bone and stress fractures. While research varies around frequency, it is estimated that the prevalence of osteopenia is between 22 and 50%, while the prevalence of osteoporosis is between 0 and 13% in able-bodied female athletes [39].

BMD declines drastically during acute phase post SCI and then continues to decline at a slower rate long-term. Osteoporosis is common amongst individuals with SCI due to reduced skeletal loading experienced over time [51]. Compared with males, females with SCI are at an even greater risk of developing osteoporosis due to progressive decline in bone mass associated with estrogen loss with aging in addition to the decline in BMD related to SCI [52]. Loss of BMD in the proximal femur occurs within 1-year post injury and can reach a fracture threshold, thus increasing risk of fracture by year 1–5 [1]. Fractures most often occur in the distal femur and proximal tibia. While the length of time post injury can greatly influence BMD, it can also be impacted by other factors such as individuals’ bone health prior to injury, weight-bearing status after the onset of injury, and region of the body. Dauty et al. found that individuals who had experienced an SCI at least one year ago experienced a decrease in distal femur BMD of 52% and proximal tibia BMD of 70% [53]. While it is well known that individuals with SCI experience significant loss of overall BMD [35,51,54,55], there is little research looking more specifically at BMD amongst athletes with SCI (Table 5).

While overall BMD is generally lower, some early research suggests that individuals with SCI may have equal or greater lumbar BMD compared to the able-bodied population [51,56]. While the exact mechanism for this is still debated, it is thought to be due to continued loading on this region and effects of extended duration in a seated posture on the spine [56]. However, more recent research has challenged this idea and actually suggests that the use of DXA in this population may overestimate BMD in the spine due to the calcification associated with moderate to high degenerative joint disease (DJD) [55,57]. Bauman et al. [55] measured BMD using two assessment tools: DXA and quantitative computerized tomography (qCT). They found that subjects with moderate/high DJD had significantly higher *T*-scores using DXA compared to qCT. There was no difference found in individuals with mild DJD [55]. Therefore, individuals with SCI with moderate to high DJD may be at a greater risk for fracture and have a lower lumbar BMD than initially assumed.

It is widely accepted that regular physical activity can help to maintain and potentially improve bone density in able-bodied persons. However, this research has primarily been done in able-bodied individuals versus para athletes. Miyahara et al. [54] observed 28 male wheelchair athletes with SCI and found that while BMD in the legs, trunk, and entire body was negatively associated with the time period since injury, the sooner that the para athlete returned to a sport post injury, the higher the BMD in those regions. Compared to able-bodied athletes, the para athletes with SCI were found to have lower BMD in their legs and whole body, although they were found to have greater BMD in their arms. The greater BMD in the arm region suggests that increased loading to the arms through physical activity may help to preserve bone mass in the radial density in athletes with SCI [54,56], although overall BMD continues to be lower in para athletes compared to able bodied athletes.

Another study examining male paraplegic wheelchair basketball players with SCI found a greater distal radial density in the athletes compared with sedentary paraplegic individuals with SCI [56]. There was no difference found in lumbar and hip densities between groups, which suggests that while the athletes experienced a change in BMD above the level of injury, there was no change experienced below the site of injury amongst this population. While this research is limited, it suggests that athletes with SCI may have improved BMD in the distal radius compared to sedentary individuals with SCI. However, the BMD of athletes with SCI was not compared to that of able-bodied athletes, and thus it is unknown how these results compare.

This preliminary research suggests that engaging in physical activity may be extremely beneficial in helping to slow the acceleration of bone loss in people with SCI. Athletes with SCI have a lower total body BMD compared to able-bodied athletes; however, they may have greater BMD above the level of lesion compared with sedentary individuals with SCI [56]. To date, no research has been conducted around BMD amongst female athletes, and thus more research is warranted in order to make this conclusion, particularly amongst female athletes with a SCI.

Athletes with SCI may also be at increased risk of low BMD due to low micronutrient intake and nutrient deficiencies [37,58]. Krempien and Barr [9] found that athletes with SCI tended to have inadequate intake of certain micronutrients associated with bone health including calcium and vitamin D. The inclusion of supplements increased the overall intake of these nutrients; however, athletes still fell below the recommended dietary intake for both of these micronutrients [9]. Other studies have found similar results in that athletes with SCI failed to meet the Estimated Average Requirement (EAR) for calcium, magnesium, and vitamin D [8,11]. This is of particular concern given that athletes with SCI have a greater risk of low BMD, and these specific nutrients play a key role in bone health [58]. Finally, given that low BMD is common in most individuals with SCI, regardless of energy intake, diagnostic criteria may need to be altered for assessing risk of LEA in athletes with SCI.

## 4. Discussion

Much of the current research around EA, nutrient issues, and the prevalence of LEA has been conducted in able-bodied athletes. However, athletes with SCI have unique physical challenges and health issues, which may put them at increased risk for low nutrient intake and LEA. With the exception of vitamin D, the risk of nutrient deficiencies is unknown in this population. In addition, no research has been done around EA in athletes with SCI. Thus, recommendations around energy needs and risk for LEA amongst athletes with disabilities is unknown. Furthermore, the criteria used to assess LEA among athletes with SCI may need to be altered given that low BMD is common in most individuals with SCI, regardless of energy intake. Despite these challenges, as the number of US Paralympic athletes continues to grow, research is warranted for supporting this population of athletes in providing assessment and treatment recommendations for the sports medicine team (physicians, trainers, coaches, dietitians, and other personnel) and coaches.

## 5. Conclusions 

In conclusion, athletes with SCI may be at increased risk around inadequate nutrient intake and LEA. However, the findings of this review should be weighed with limitations as there are no current studies which assess EA amongst this population. Due to this limitation, the authors used research which assessed nutrient and energy intake amongst para athletes and sedentary individuals. As the number of para athletes continues to grow, research and understanding around the risk of low nutrient intake and LEA in this population is necessary.

## Figures and Tables

**Table 1:** Overview of studies that have examined energy expenditure amongst para athletes including athletes with SCI.

Reference	Subjects	Methods	Results
Buchholtz et al. [18]	34 control 28 paraplegics with SCI	indirect calorimetry following 12-h fast	RMR C: 1676 ± 223 kcal/day RMR SCI: 1472 ± 228 kcal/day (No diff. when adjusted for FFM) TEF C: 6.25 ± 2.2% TEF SCI: 5.53 ± 1.8%
Pelly et al. [20]	7 M WC athletes with SCI and 6 able-bodied controls	indirect calorimetry following 12-h fast	RMR no difference found between groups When adjusted for LTM: SCI: 35 ± 7 kcal/kg LTM C: 30 ± 2 kcal/kg LTM Prediction equations—not warranted for SCI population

SCI = spinal cord inury, RMR = resting metabolic rate, C = control subjects, FFM fat free mass, TEF = thermic effect of feeding, WC = wheelchair, LTM = lean tissue mass.

**Table 2:** Research examining risk of disordered eating and eating behaviors amongst para athletes, including athletes with SCI.

Reference	Subjects	Methods	Results
Krempien et al. [34]	32 M/F athletes w/SCI	TFEQ	M—strong tendency towards cognitive restraint M/F—lower scores for disinhibition and hunger
Blauwet et al. [35]	248 M/F para athletes	Online questionnaire	40% reported currently trying to lose wt 61% reported attempting to change body composition

Male = male, F = female, SCI = spinal cord injury, TFEQ = Three Factor Eating Questionnaire, Wt = weight.

**Table 3:** Overview of studies examining macronutrient intake of para athletes.

Reference	Subjects	Methods	Results-Macronutrients
Gerrish et al. [8]	39 M/F SCI athletes	Self-reported, single 24-h food journal in autumn and winter	Fall: Energy (M): 1893 ± 725 kcal/day Energy (F): 1602 ± 718 kcal/day CHO (M/F): 3.5 ± 1.17 g/kg Pro (M/F): 1.4 ± 0.2 g/kg Winter: Energy (M): 1,669 ± 683 kcal/day Energy (F):1463 ± 844 kcal/day CHO (M/F): 3.1 ± 0.8 g/kg Pro (M):1.2 ± 0.4 g/kg
Krempien et al. [9]	32 M/F SCI athletes	3-day self-reported food journal kept at home and training camp	Energy (M): 2156 ± 431 kcal/day Energy (F): 1991 ± 510 kcal/day CHO (M/F): 4.4 ± 1.1 g/kg Pro (M/F):1.4 ± 0.4 g/kg
Grams et al. [11]	17 M WC BB athletes	3-day weighted food journal over 3 consecutive days during 3 training camps over 2 consecutive years.	Energy: 2673 ± 485 kcal/day CHO: 3.9 (1.8; 8.1) g/kg Pro: 1.7 ± 0.6 g/kg
Eskiki and Ersoy [25]	22 F WC athletes	24-h retrospective diet recall	Energy: 2867.8 ± 23.6 kcal/day CHO: 5.3 ± 1.5 g/kg Pro: 1.6 ± 0.3 g/kg
Madden et al. [16]	40 M/F Paralympic athletes	3-day, consecutive self-reported food journal	Energy (M): 2092 (1695–2690) kcal/day Energy (F): 1602 (1439–2059) kcal/day CHO (M/F): 3.5 ± 1.1 g/kg Pro (M/F): 1.5 (1.3–1.7) g/kg

Intakes are presented as mean and standard deviation or mean and range. M = males, F = females, SCI = spinal cord injury, Energy = energy intake, CHO = carbohydrate, Pro = protein, WC = wheelchair, BB = basketball, TEE = total energy expenditure.

**Table 4:** Overview of studies examining micronutrient intake of para athletes, including athletes with SCI.

Reference	Subjects	Methods	Reference Tool	Results-Micronutrients
Gerrish et al. [8]	39 M/F SCI athletes	Self-reported, single 24-h food journal in autumn and winter	EAR	Low: vitamin B6, vitamin B12, vitamin C, folate, calcium, iron, magnesium, zinc
Krempien et al. [9]	32 M/F para athletes	3-day, self-reported food journal kept at home and training camp	EAR	Low: (M)-calcium, magnesium, zinc, riboflavin, folate, vitamin B12, vitamin D (F)-calcium, magnesium, folate, vitamin D
Goosey-Tolfrey [10]	23 M/F WC para athletes	7-day consecutive, participant-reported weighed food journal	UK DRV	Low: iron, fiber
Grams et al. [11]	17 M WC BB para athletes	3-day food weighed journal over 3 consecutive days during 3 training camps in 2 consecutive years	RDA	Low: vitamin E, calcium
Eskiki and Ersoy [25]	22 F WC para athletes	24-h retrospective diet recall	RDA	Low: vitamin B1, folic acid, magnesium, iron, fiber
Madden et al. [16]	40 M/F Paralympic athletes	3-day consecutive, self-reported food journal	RDA	Low: (M): vitamin A, folate (F): iron, calcium

M = male, F = female, SCI = spinal cord injury, EAR = Estimated Average Requirement, DRI = Dietary Reference Intake, UK DRV = UK Dietary Reference Value, WC = wheelchair, BB = basketball, RDA = Recommended Dietary Allowance.

**Table 5:** Overview of studies examining BMD in para athletes.

Reference	Subjects	Methods	Results
Miyahara et al. [54]	28 M WC athletes w/SCI 25 M AB athletes	DXA	-No significant difference in BMD based on level of injury, sport, age amongst SCI. -BMD in trunk, legs, & whole body negatively associated w/time since injury. -Sooner SCI returned to sport post injury, greater the BMD in trunk, legs, and whole body. -Greater BMD in arms and lower BMD in legs and whole body of SCI compared with AB.
Goktepe et al. [56]	17 M WC BB athletes w/SCI 17 sedentary WC w/SCI	DXA	-Greater radial density in WC BB compared to sedentary WC. -No diff in lumbar and hip BMD between WC BB and sedentary WC.

M = male, WC = wheelchair, SCI = spinal cord injury, AB = able-bodied, DXA = dual-energy X-ray absorptiometry, BMD = bone mineral density, BB = basketball.
